# Sniffing oxytocin: Nose to brain or nose to blood?

**DOI:** 10.1038/s41380-023-02075-2

**Published:** 2023-04-25

**Authors:** Shuxia Yao, Yuanshu Chen, Qian Zhuang, Yingying Zhang, Chunmei Lan, Siyu Zhu, Benjamin Becker, Keith M. Kendrick

**Affiliations:** 1 Center of Psychosomatic Medicine, Sichuan Provincial Center for Mental Health, Sichuan Provincial People’s Hospital, University of Electronic Science and Technology of China, Chengdu, China; 2 The MOE Key Laboratory for Neuroinformation, School of Life Science and Technology, University of Electronic Science and Technology of China, Chengdu, China; 3 Center for Cognition and Brain Disorders, The Affiliated Hospital of Hangzhou Normal University, Hangzhou, Zhejiang Province China; 4 Department of Molecular Psychology, Institute of Psychology and Education, Ulm University, Ulm, Germany

**Keywords:** Neuroscience, Drug discovery

## Abstract

In recent years ample studies have reported that intranasal administration of the neuropeptide oxytocin can facilitate social motivation and cognition in healthy and clinical populations. However, it is still unclear how effects are mediated since intranasally administered oxytocin can both directly enter the brain (nose to brain) and increase peripheral vascular concentrations (nose to blood). The relative functional contributions of these routes are not established and have received insufficient attention in the field. The current study used vasoconstrictor pretreatment to prevent intranasal oxytocin (24 IU) from increasing peripheral concentrations and measured effects on both resting-state neural (electroencephalography) and physiological responses (electrocardiogram, electrogastrogram and skin conductance). Results demonstrated that intranasal oxytocin alone produced robust and widespread increases of delta-beta cross-frequency coupling (CFC) from 30 min post-treatment but did not influence peripheral physiological measures. As predicted, vasoconstrictor pretreatment greatly reduced the normal increase in peripheral oxytocin concentrations and, importantly, abolished the majority of intranasal oxytocin effects on delta-beta CFC. Furthermore, time-dependent positive correlations were found between increases in plasma oxytocin concentrations and corresponding increases in delta-beta CFC following oxytocin treatment alone. Our findings suggest a critical role of peripheral vasculature-mediated routes on neural effects of exogenous oxytocin administration with important translational implications for its use as an intervention in psychiatric disorders.

## Introduction

Over the last few decades, the hypothalamic neuropeptide oxytocin (OXT) has been at the forefront of interest in human neuropsychopharmacology and social neuroscience research with many studies reporting an important modulatory role in social motivation and cognition [1–3]. From a translational point of view the therapeutic potential of targeting the OXT signaling systems has received some support from clinical trials such as in autism, Prader-Willi syndrome and schizophrenia [4–9], although findings have been variable [10–12]. One of the key unresolved questions is the mechanism of action whereby exogenously administered OXT produces its modulatory effects on brain and behavior. Going forward, it is important to establish the route(s) whereby OXT produces its functional effects, especially in the context of its potential therapeutic use.

While, there is a widely distributed network of OXT projections and receptors in the brain [13, 14], early animal model research demonstrated that the blood-brain-barrier (BBB) was only weakly permeable to peripherally administered OXT (<0.1%) [15, 16]. In human studies this issue was addressed by using an intranasal administration route inspired by findings that neuropeptides could enter the cerebrospinal fluid as well as the peripheral vascular system via this route [17–19]. While there was initially some debate as to whether intranasal administration could result in OXT entering the brain directly [20, 21], a number of animal model studies have subsequently confirmed that it may do so via an extracellular route involving the olfactory and trigeminal nerves or perineural clefts in the nasal epithelium [22], thereby bypassing the BBB [23, 24].

While it has widely been assumed intranasal OXT produces its effects on brain and behavior via a direct route this is increasingly being questioned. Following intranasal administration, OXT is also absorbed by blood vessels in the nasal mucosa and animal studies have reported functional effects of OXT administered via peripheral routes which do not permit direct entry into the brain (i.e., intravenous, subcutaneous and intraperitoneal) [23]. Furthermore, some early studies on autistic individuals reported behavioral effects of intravenous administration [25, 26] and intravenous and intranasal OXT have similar effects on regional brain activity [27]. The effects of orally administered OXT have also been increasingly investigated in humans. An initial study comparing intranasal and oral administration of OXT (24 IU) found that the oral dose produced greater responses to emotional faces in brain reward areas. On the other hand, while intranasal OXT decreased amygdala responses to emotional faces, oral OXT had the opposite effect [28]. However, two subsequent studies have found identical effects of intranasal and oral OXT on both visual attention and reducing state anxiety [29, 30]. Thus, there is evidence for both route-dependent and route-independent effects of OXT and further studies are urgently needed to disentangle their respective contribution.

One approach to disentangle some of the above questions is to restrict intranasal OXT to only produce potential functional effects via direct entry into the brain while at the same time preventing it from entering the peripheral circulation. A study on rats reported that this could be achieved for other peptides using pre-treatment with the vasoconstrictor (VC), phenylephrine [31]. In the present study in humans we therefore adopted a similar strategy but using a more widely used VC, xylometazoline [32].

The next important question is choice of potential biomarkers for both neural and peripheral functional effects of OXT. While previous studies have tended to focus on either task-based or resting-state fMRI responses to OXT [2, 3, 33], going forward, and to facilitate simultaneous blood sampling and taking physiological measures, another option is to capitalize on resting-state electroencephalography (rsEEG). To date, relatively few studies have studied effects of intranasal OXT on rsEEG with some evidence for effects on microstates [34, 35]. Other promising candidates are measures of neural synchronization between alpha and theta rhythms [36] and also altered delta-beta cross frequency coupling (CFC) [37]. Delta-beta coupling is one of the most studied CFCs between slow and fast oscillations and proposed to reflect subcortical-cortical crosstalk, particularly the interplay between emotional/motivational systems and cognition [38–40]. As such, the functional role of delta-beta CFC corresponds well to the demonstrated role of OXT in modulating social cognition/motivation and emotion [1, 41, 42]. For peripheral vagal/parasympathetic markers of OXT effects the most studied are altered heart rate variability (HRV) from the electrocardiogram (ECG) and skin conductance response (SCR) [27, 36, 43–46], although gastrointestinal effects of intravenous OXT have also been reported [47] and thus alterations in electrogastrogram (EGG) activity might also reflect vagally-mediated effects.

Against this background the current study compared delta-beta CFC, ECG, SCR and EGG responses as well as changes in plasma OXT concentrations following a single dose of intranasal 24 IU OXT either following pre-treatment with placebo (PLC) or the VC and compared to individuals receiving PLC-controlled treatment. We first hypothesized that VC pretreatment would prevent intranasal OXT from increasing plasma concentrations. We next hypothesized that if neural effects of intranasal OXT were mediated by direct entry into the brain, then VC pretreatment would have no effect on delta-beta CFC, although parasympathetic/vagal physiological changes would not occur. If, on the other hand, effects were mediated by increased peripheral concentrations then VC pretreatment would prevent both neural and autonomic effects.

## Methods and materials

### Participants and treatment

96 healthy adult male subjects were recruited for the present study and were instructed to abstain from consuming alcohol and caffeine during the 24 h before the experiment. Based on similar previous studies (e.g., [27, 48]) and a priori power analysis for a mixed ANOVA using the G*Power v.3.1 toolbox [49] with a power > 0.8 (effect size = 0.25, α = 0.05), this sample size was adequate to detect reliable OXT effects on changes of blood concentrations and delta-beta CFC. Subjects were randomly assigned to one of three treatments. For VC pre-treatment, individuals in all three treatment groups received both a pretreatment (either VC or PLC) followed 10 min later by a main treatment (either OXT or PLC). Thus, one group first received intranasal VC (4 sprays; Otrivin 0.1% nasal spray, Novartis, Switzerland) followed 10 min later by intranasal administration of OXT (VC + OXT group—24 IU; delivered as 6, 0.1 ml puffs of 4 IU—Oxytocin Spray, Sichuan Defeng Pharmaceutical Co. Ltd, China). A second group received a PLC control for VC (4 sprays of 0.9% saline) followed by intranasal OXT (PLC + OXT group—6 sprays of 4 IU) and a third group received VC pretreatment (4 sprays of 0.1% Otrivin) followed by the PLC control for OXT (VC + PLC group—6 sprays of PLC, i.e., 0.9% saline and glycerin but without the peptide). Five subjects were excluded due to failure of blood sampling (3 subjects), falling asleep (1 subject), or technical problems during data recording (1 subject). Consequently, 31 subjects in the PLC + OXT group, 30 subjects in the VC + OXT group, and 30 subjects in the VC + PLC group were included in the final analysis. In interviews at the end of the study subjects were unable to identify better than chance whether they had received VC or PLC pretreatment (*χ*^2^ = 2.47, *p* = 0.116) or subsequent OXT or PLC treatment (*χ*^2^ = 0.28, *p* = 0.600). Subjects completed Chinese versions of validated questionnaires of mood and personality traits before treatment to control potential confounding effects (details see Supplementary Information—SI). All procedures in the present study were in accordance with the latest version of the Declaration of Helsinki and were approved by the local ethical committee of University of Electronic Science and Technology of China. This study was pre-registered at clinical trials.gov (ID: NCT04134663). Written informed consents were obtained from all subjects before study inclusion.

### Experimental protocol

**Fig. 1:**
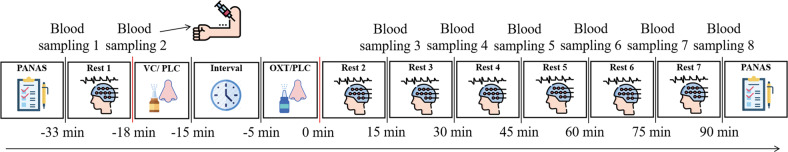
Timeline of experimental protocol. A randomized, placebo-controlled, double-blind, between-subject design was used in the present study. For VC pre-treatment, participants in all three treatment groups received both a pretreatment (either VC or PLC) followed 10 min later by a main treatment (either OXT or PLC). Thus, one group first received intranasal VC followed 10 min later by intranasal administration of OXT (VC + OXT group). A second group received a PLC control for VC followed by intranasal OXT (PLC + OXT group) and a third group received VC pretreatment followed by the PLC control for OXT (VC + PLC group). 31 subjects in the PLC + OXT group, 30 subjects in the VC + OXT group, and 30 subjects in the VC + PLC group were included in the final analysis. Blood samples were collected every 15 min before and after treatments for measuring changes of plasma oxytocin concentrations. 8 blood samples were collected in total with blood sample 1 and 2 collected before treatment serving as baseline. During each 15 min interval, resting state EEG and other physical responses were recorded (Rest 1-7). The two boundary lines in red indicate the start and end of the treatments. PANAS Positive and Negative Affect Schedule. OXT oxytocin. VC vasoconstrictor. PLC placebo.

A randomized, placebo-controlled, double-blind, between-subject design was used in the present study (see Fig. 1 for a complete timeline). Eight blood samples (5 ml) were collected into 6 ml ETDA tubes using an indwelling venous catheter for OXT concentration analyses (details see SI). The first 2 blood samples were collected before treatment with a 15 min interval (Rest 1) and served as baseline. To ensure effective nasal blood vessel vasoconstriction, subjects were instructed to self-administer VC or PLC 10 min before OXT/PLC treatment. 2 puffs of VC or PLC were administered in each nostril alternately with a 30 s interval. After 10 min, they were asked to self-administer 6 puffs of OXT or PLC in alternate nostrils following a standardized protocol [50]. Another 6 blood samples (blood samples 3–8) were then collected with each sample being taken every 15 min. During each 15-min resting-state interval (Rest 1–7), subjects were instructed to sit quietly, stay relaxed, focus on a white fixation presented on a black background and think of nothing in particular. 5-min data of rsEEG and other physiological measurements recorded at the end of each 15-min interval were extracted for statistical analyses. To avoid possible circadian influences, the experiment started at around 14.00 h in a quiet testing room and lasted approximately 3 h including experimental preparation.

### Data acquisition and analyses

#### RsEEG recording and data processing

The rsEEG was recorded at a sampling rate of 500 Hz using a 64-channel actiCHamp system (Brain Products GmbH, Germany). Offline EEG data were preprocessed using the EEGLAB v2019.0 toolbox [51]. Averaged spectral power values were calculated for delta (1–4 Hz), low beta (beta_low_, 13–25 Hz), and high beta (beta_high_, 25-35 Hz) frequencies respectively. Power values were further averaged across electrodes for prefrontal (Fp1, Fp2), frontal (F3, F4, Fz), central (C3, C4, Cz), parietal (P3, P4, Pz), temporal (T7, T8), and occipital (O1, O2, Oz) regions based on the 10–20 electrode system (see Supplementary Fig. S1). Power-based connectivity was then computed between each pair of the 6 regions (inter-regional CFC) and within each region (intra-regional CFC) following guidelines in Cohen (2014) [52]. This led to 6 × 5 = 30 pairs of inter-regional delta-beta_low_/beta_high_ CFC and 6 pairs of intra-regional and delta-beta_low_/beta_high_ CFC. Correlation coefficients were calculated using the Spearman correlation and were Fisher-Z transformed for subsequent statistical analyses [52]. Phase-amplitude coupling (PAC) was also analyzed by applying the traditional method introduced by Canolty et al. (details see SI) [53].

#### Physiological data recording and processing

Physiological measurements were recorded at a sampling rate of 1000 Hz using a BIOPAC MP150 system (BIOPAC Systems, Inc.) in accordance with the BIOPAC manual. Physiological data was processed using AcqKnowledge v4.4 software following the manual or previous studies. The resultant SCR data was log-transformed and the averaged data was extracted for subsequent statistical analyses. Consistent with previous studies examining OXT effects on HRV [46, 54], mean heart rate, high frequency HRV, the detrended fluctuation scaling exponent (DFAα1) were extracted to test for treatment effects. Logarithmic transformation was applied where variables were not normally distributed. For EGG, mean amplitude and cycles per min were exported for subsequent analyses (details see SI).

### Statistical analyses

To determine treatment effects on changes of blood OXT concentrations, delta and beta power, delta-beta CFC, and peripheral responses (SCL, ECG, and EGG), repeated-measures ANOVAs were applied on indices of these measurements with timepoint (baseline-90 min for OXT concentrtaions and Rest 1–7 for other measurememts) as within-subject factor and treatment (PLC + OXT vs. VC + OXT vs. VC + PLC) as between-subject factor. Greenhouse-Geisser correction was used when sphericity assumptions were violated. Bonferroni-correction was employed for multiple comparisons in post-hoc analyses. For CFC, we first examined treatment effects on global CFC by averaging across all the CFC pairs. The global CFC was then disentangled into inter- and intra-regional CFC to determine whether treatment effects varied across them. Treatment effects on indiviudal CFC were also reported for inter- and intra-regional CFC seperately. Given the non-normal distribution of plasma OXT concentrations, Spearman correlation coefficients were used to calculate correlations between changes of delta-beta CFC and plasma OXT concentrations.

## Results

### Demographics and questionnaires

One-way ANOVA revealed no significant group differences of age, mood and personality trait scores (ps > 0.131; Supplementary Table S1). Pairwise comparisons between any of the two treatments further confirmed no significant group differences (ps > 0.186).

### Plasma OXT concentration changes

**Fig. 2:**
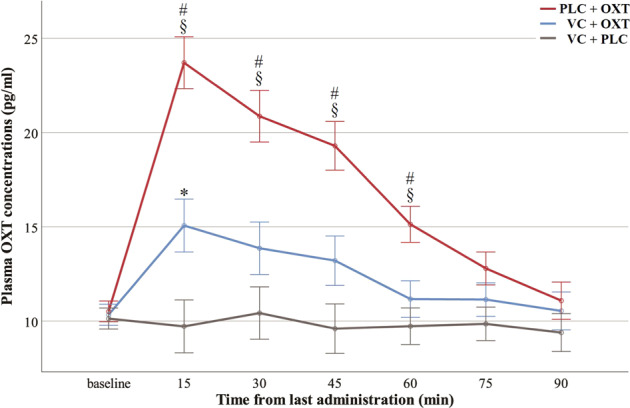
Pharmacokinetic profiles of raw plasma oxytocin concentrations over time for each treatment. Baseline is an average of concentrations in blood samples 1 and 2. The remaining timepoints (15–90 min) correspond to blood samples 3-8. Data presented are mean ± standard error. “§” indicates significantly higher oxytocin concentrations following PLC + OXT in comparison to VC + PLC (*p* < 0.05 FWE corrected). “#” indicates significantly higher oxytocin concentrations following PLC + OXT in comparison to VC + OXT (p < 0.05 FWE corrected). “*” indicates significantly higher oxytocin concentrations following VC + OXT in comparison to VC + PLC (*p* < 0.05 FWE corrected). OXT oxytocin. VC vasoconstrictor. PLC placebo.

Raw plasma OXT concentration changes over time are presented in Fig. 2. A repeated-measures ANCOVA on raw plasma OXT concentrations after treatment with OXT concentrations at baseline as a covariate showed a significant main effect of treatment (F(2, 87) = 19.71, *p* < 0.001, *η*_p_^2^ = 0.312). Importantly, the interaction between timepoint and treatment was significant (*F*(8.10, 352.34) = 8.62, *p* < 0.001, *η*_p_^2^ = 0.165). Post-hoc analyses revealed significantly higher OXT concentrations from 15 to 60 min following PLC + OXT in comparison to VC + OXT (15 min: *p* < 0.001; 30 min: *p* = 0.001; 45 min: *p* = 0.003; 60 min: *p* = 0.012) and VC + PLC treatments (all ps < 0.001). However, following VC + OXT treatment OXT concentrations were higher than after VC + PLC treatment at 15 min post-treatment around its peak in blood sample 3 (*p* = 0.024), but not at other timepoints (all ps ≥ 0.162). Plasma OXT concentration changes in ratios relative to baseline over time are presented in the Supplementary Fig. S2 and showed a highly similar pattern to raw concentrations except that there were no siginificant differences over time between VC + OXT and VC + PLC treatments (ps ≥ 0.123).

### Delta-beta_low_ CFC

**Fig. 3:**
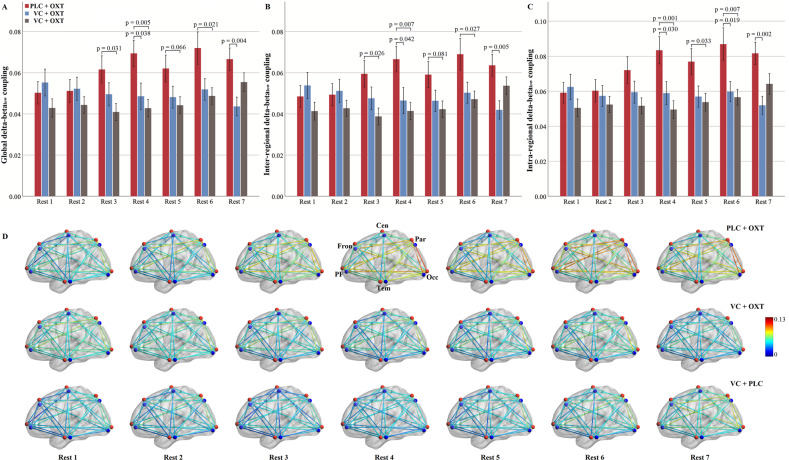
Comparisons of averaged and individual delta-beta_low_ cross-frequency coupling over timepoints for each treatment. Comparisons of global (**A**), inter-regional (**B**) and intra-regional (**C**) delta-beta_low_ cross-frequency coupling over timepoints for each treatment. **D** Strengths of each inter- and intra-regional delta-beta_low_ CFC from Rest 1 to 7. Treatment effects on delta-beta_low_ CFC and its interaction with timepoint were examined using repeated-measures ANOVAs. *P* values in (**A**–**C**) are from post-hoc analyses disentangling significant interaction effects between treatment and timepoint. While the red sphere in (**D**) indicates delta frequency, the blue sphere indicates low beta frequency for each region. Histograms show mean and standard error. OXT oxytocin, VC vasoconstrictor, PLC placebo.

Repeated-measures ANOVAs were employed on strengths of global delta-beta_low_ CFC and revealed significant main effects of timepoint (*F*(5.06, 444.83) = 2.66, *p* = 0.021, *η*_p_^2^ = 0.029) and treatment (*F*(2, 88) = 3.46, *p* = 0.036, *η*_p_^2^ = 0.073). Importantly, the interaction between timepoint and treatment was significant (F(10.11, 444.83) = 3.73, *p* < 0.001, η_p_^2^ = 0.078). Post-hoc analyses showed consistently stronger global delta-beta_low_ CFC following PLC + OXT in comparison to VC + PLC treatments from Rest 3 to 6 (all ps ≤ 0.031), except that this effect was marginal in Rest 5 (*p* = 0.066). Furthermore, delta-beta_low_ CFC was also stronger following PLC + OXT relative to VC + OXT treatment in Rest 4 (*p* = 0.038) and 7 (*p* = 0.004) (Fig. 3A). However, no significant CFC differences were found between VC + OXT and VC + PLC treatments (all ps ≥ 0.274). Disentangling the global CFC into inter-regional and intra-regional CFC revealed a highly similar pattern of inter- (Fig. 3B) and intra-regional delta-beta_low_ CFCs (Fig. 3C; see Supplementary Table S2 for statistics).

Strengths of each inter- and intra-regional delta-beta_low_ CFC over timepoint were presented in Fig. 3D. Repeated-measures ANOVAs revealed a generally similar pattern of individual regional CFCs across treatments over timepoints to the global one (see Supplementary Figs. S3A, S4 for statistics and patterns of each pair respectively).

### Delta-beta_high_ CFC

**Fig. 4:**
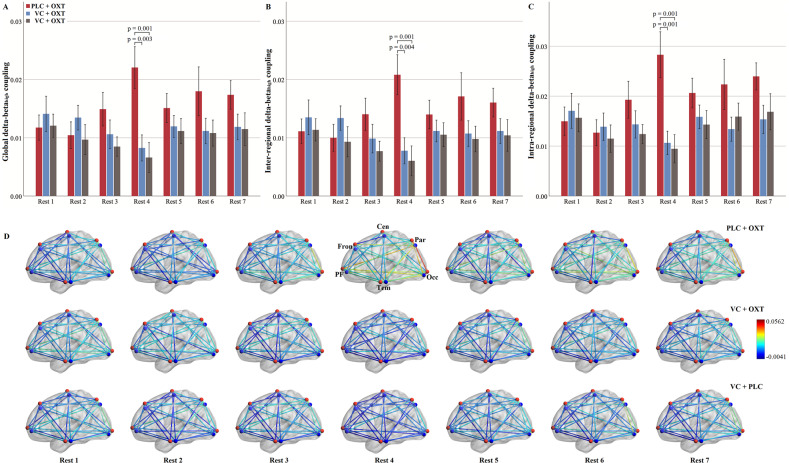
Comparisons of averaged and individual delta-beta_high_ cross-frequency coupling over timepoints for each treatment. Comparisons of global (**A**), inter-regional (**B**) and intra-regional (**C**) delta-beta_high_ cross-frequency coupling over timepoints for each treatment. **D** Strengths of each inter- and intra-regional delta-beta_high_ CFC from Rest 1 to 7. Treatment effects on delta-beta_high_ CFC and its interaction with timepoint were examined using repeated-measures ANOVAs. *P* values in (**A**–**C**) are from post-hoc analyses disentangling significant interaction effects between treatment and timepoint. While the red sphere in (**D**) indicates delta frequency, the blue sphere indicates high beta frequency for each region. Histograms show mean and standard error. OXT oxytocin, VC vasoconstrictor, PLC placebo.

For global delta-beta_high_ CFC, there was only a significant interaction effect between timepoint and treatment (F(10.09, 444.01) = 2.43, *p* = 0.008, *η*_p_^2^ = 0.052). Post-hoc analyses revealed significantly stronger global delta-beta_high_ CFC following PLC + OXT relative to VC + PLC (*p* = 0.001) and VC + OXT (*p* = 0.003) treatments in Rest 4 (Fig. 4A). In contrast, no significant CFC differences were found between VC + OXT and VC + PLC treatments (*p* > 0.999). Separate examination of inter-regional and intra-regional delta-beta_high_ CFC showed an identical pattern of inter- (Fig. 4B) and intra-regional (Fig. 4C) delta-beta_high_ CFC to the global effect (see Table S2 for statistics).

Strengths of each inter- and intra-regional delta-beta_high_ CFC over timepoint were presented in Fig. 4D. Repeated-measures ANOVAs revealed a generally similar pattern of individual CFC across treatments over timepoints to the global one (see Supplementary Figs. S3B, S5 for statistics and patterns of each pair respectively).

### Associations between plasma OXT concentrations and CFC

**Fig. 5:**
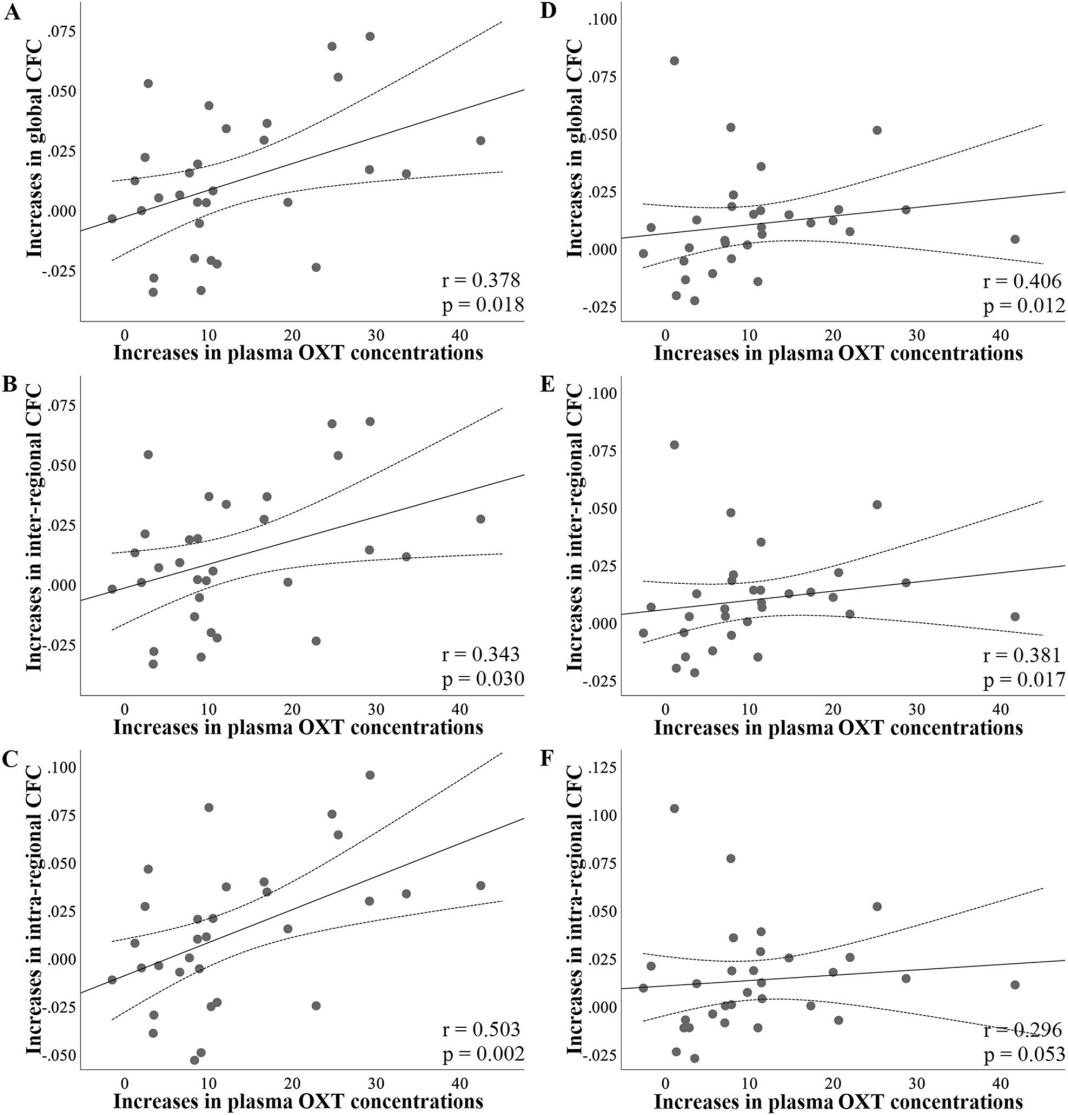
Scatter plots and results of correlations between changes in plasma oxytocin (OXT) concentrations and delta-beta CFC following placebo (PLC) + OXT treatment. **A** Absolute increases in plasma OXT concentrations in blood sample 3 relative to baseline were positively correlated with corresponding increases in global delta-beta_low_ CFC in Rest 3 relative to baseline. Similar positive correlations were found for inter- (**B**) and intra-regional (**C**) delta-beta_low_ CFC in Rest 3. **D** Absolute increases in plasma OXT concentrations in blood sample 4 relative to baseline were positively correlated with corresponding increases in global delta-beta_high_ CFC in Rest 4 relative to baseline. Similar positive correlations were found for inter- (**E**) and intra-regional (**F**) delta-beta_high_ CFC in Rest 4. Spearman correlation coefficients were used to calculate these correlations. Changes in OXT concentrations are in pg/ml. The dotted line represents the 95% confidence interval.

We examined whether there were time-dependent positive correlations (one-tailed) between increases of plasma OXT concentrations following treatment (difference scores relative to baseline) and the enhancement effect of OXT on delta-beta CFC (difference scores relative to CFC in Rest 1). For delta-beta_low_ CFC, a significant positive correlation was found between increased plasma OXT concentrations of blood sample 3 and corresponding increased global delta-beta_low_ in Rest 3 (*r* = 0.378, *p* = 0.018; Fig. 5A) only following PLC + OXT treatment, with similar positive correlations for inter- (*r* = 0.343, *p* = 0.030; Fig. 5B) as well as intra-regional delta-beta_low_ CFC (*r* = 0.503, *p* = 0.002; Fig. 5C). However, there were no significant correlations following either VC + OT (all ps ≥ 0.326) or VC + PLC treatments (all ps ≥ 0.233). For other timepoints, there were similar trends of positive correlations in Rest 4 (*r* = 0.252, *p* = 0.085) and Rest 5 (*r* = 0.281, *p* = 0.063) following PLC + OXT treatment, although they were not significant.

Given that treatment effects were exclusively found in Rest 4 (45 min post-treatment) for delta-beta_high_ CFC, the correlation analysis was restricted to Rest 4. Results showed a significant positive correlation between increased plasma OXT concentrations in blood sample 4 and corresponding increased global delta-beta_high_ in Rest 4 (*r* = 0.406, *p* = 0.012; Fig. 5D) following PLC + OXT treatment. Separate examination of inter- and intra-regional delta-beta_high_ CFC showed a significantly positive correlation for inter- (*r* = 0.381, *p* = 0.017; Fig. 5E) but a marginal one for intra-regional (*r* = 0.296, *p* = 0.053; Fig. 5F) delta-beta_high_ CFC. There were also similar trends of positive correlations in Rest 4 (global: *r* = 0.243, *p* = 0.098; inter-regional: *r* = 0.277, *p* = 0.069) following VC + OT but not VC + PLC treatments (all ps ≥ 0.128). There were no significant correlations between plasma OXT concentrations and delta-beta_low_/beta_high_ CFC across groups at baseline (all ps ≥ 0.163).

### Delta and beta_low_/beta_high_ power and delta-beta PAC

Repeated-measures ANOVAs on power values of delta, beta_low_, and beta_high_ bands revealed no significant effects related to treatment (all ps ≥ 0.141). For delta-beta_low_/beta_high_ PAC, there were also no significant treatment effects (all ps ≥ 0.149; details see SI).

### Parasympathetic/vagal indices

Repeated-measures ANOVAs on parasympathetic/vagal indices also revealed no significant treatment effects on SCL, HRV or ECG indices (all ps ≥ 0.127; details see SI).

## Discussion

By incorporating a novel approach of including a VC treatment prior to intranasal OXT administration, the present study firstly investigated whether this was an effective strategy in blocking intranasally applied OXT from entering the peripheral circulation. Secondly, the study aimed to use this strategy to help disentangle whether functional effects of intranasal administration are mediated by direct entry of OXT into the brain as opposed to by increased peripheral concentrations. Results showed that while intranasal OXT significantly increased plasma OXT concentrations from 15 to 60 min post-treatment, VC pretreatment greatly reduced this. Intranasal OXT was found to produce robust and widespread increases in the strength of delta-beta CFC, although not in parasympathetic/vagally-mediated measures. When intranasal OXT was preceded by VC treatment only a few significant increases in delta-beta CFC were still observed. Furthermore, time-dependent positive correlations were found between increases in plasma OXT concentrations and corresponding increases in delta-beta CFC following PLC + OXT treatment. Overall, these findings using a rsEEG biomarker for effects of intranasal OXT demonstrate that while some limited effects may be due to direct entry of the peptide into the brain, the majority of observed increases in CFC appear to be mediated via increased peripheral concentrations. This finding has major potential implications for future translational research using exogenous OXT treatment strategies as an intervention.

Consistent with previous studies [19, 28, 55], plasma OXT concentrations following intranasal treatment reached a peak at 15 min post-treatment and remained significantly increased for a further 60 min compared with both the VC + PLC and VC + OXT groups. Importantly, raw plasma OXT concentrations did not differ between the latter two groups except that there was a small significant increase at 15 min post-treatment in the VC + OXT than the VC + PLC group. There were no significant differences between the two groups when using proportionate changes in concentration values. These findings indicate that VC pretreatment greatly reduced the ability of intranasal OXT to increase peripheral concentrations as planned. Thus VC acted effectively in reducing OXT absorption by nasal blood vessels in line with a previous animal study using another VC (phenylephrine) to reduce peripheral blood concentrations of other neuropeptides (hypocretin-1or the dipeptide L-Tyr-D-Arg) administered intranasally [31]. Efficacy validation of this novel treatment strategy in humans is of great therapeutic application potential for pharmaceuticals targeting the CNS, particularly those with adverse side effects of peripheral exposure. In the context of the current study, these findings provide a methodological approach for determining the relative functional contributions of substances administered intranasally in terms of being mediated via increased peripheral concentrations as opposed to direct-entry into the brain.

In terms of rsEEG-based biomarkers of intranasal OXT we found no evidence for effects on the power of different individual frequencies in agreement with several previous studies [36, 37]. We did however observe widespread and robust increases in amplitude, but not phase-amplitude, coupling between delta and beta frequencies, indicating that this may be an important biomarker for neural effects of OXT. Analyses revealed that there was a significant global increase in both delta-beta_low_ and -beta_high_ CFC in the PLC + OXT compared with the VC + PLC group. Disentanglement of the global CFC into inter- and intra-regional CFC revealed similar inter- and intra-regional CFC patterns to the global ones. More specifically at the regional level, robust enhancement effects of OXT were found for delta-beta_low_ CFC of the occipital region with the parietal, prefrontal, frontal and temporal regions and of the parietal region additionally with the prefrontal, frontal and temporal regions. A similar but less robust pattern of regional CFC changes was also found for delta-beta_high_. As such changes are occurring between widespread cortical networks subserving attentional, executive and sensory processing. However, given the low spatial resolution of the EEG technique, explanations regarding to functional implications of these regional specific delta-beta changes should be made with caution. Of note, while the enhancement effect of OXT on delta-beta_low_ CFC occurred from Rest 3 to 6 (i.e., 30-75 min post-treatment) and was strongest in Rest 4 (i.e., 45 min post-treatment) compared to the other two groups, its effects on delta-beta_high_ CFC were exclusively restricted to Rest 4. These time courses for rsEEG effects of intranasal OXT are very similar to those previously reported for changes in EEG microstate features [35] and regional cerebral blood flow changes [27, 48]. However, our findings are inconsistent with a previous study reporting that intranasal OXT decreased delta-beta CFC [37]. Differences between the findings may be explained by the fact that we only used male subjects and they used female ones. A number of studies have reported sex differences in neural responses to OXT [56–58]. Furthermore, we only carried out an eyes-open resting-state measurement and they found OXT effects in an eyes-closed but not eyes-open condition. Taken together, given that delta-beta CFC is a neural index of interplay between emotional/motivational systems and cognition [38–40], our findings were in line with the well-demonstrated role of OXT in modulating social cognition/motivation and emotion [1, 41, 42].

Importantly, in the context of the primary objective of the current study VC pretreatment eliminated the enhancement effect of intranasal OXT on increases in delta-beta CFC with the VC + OXT group not differing significantly from the VC + PLC group. This therefore suggests that if intranasally administered OXT is largely prevented from entering the peripheral circulation its effects on neural activity in terms of delta-beta CFC are minimal. Thus, peripherally-mediated routes may play a critical role for intranasal OXT in modulating delta-beta CFC. This assumption is further supported by time-dependent positive correlations between increases in plasma OXT concentrations in blood sample 3 and increases in delta-beta_low_ CFC in Rest 3 and between increases in plasma OXT concentrations in blood sample 4 and increases in delta-beta_high_ CFC in Rest 4 only following PLC + OXT treatment.

Although global increases in delta-beta CFC induced by intranasal OXT were eliminated by VC, analyses at a regional level revealed some evidence for changes which were maintained after it. Significant increases of delta-beta_low_ CFC following VC + OXT relative to VC + PLC treatment was found in 3 out of the 30 inter-regional links involving central-parietal, temporal-frontal and temporal-occipital pairs (2 pairs in Rest 2 and one in Rest 3, i.e., 15 and 30 min post-treatment) and in 2 pairs of delta-beta_high_ CFC involving frontal-central and frontal-prefrontal pairs (one in Rest 2 and 5 respectively, i.e., 15 and 60 min post-treatment; details see SI). Thus, these findings suggest that some changes in beta-delta CFC may have been due to direct-entry of OXT into the brain following intranasal administration. However, since we also found some evidence for an initial small increase in plasma OXT concentrations in the VC + OXT group it is possible that observed CFC changes could have been partially contributed to by them rather than entirely by direct entry into the brain. Interestingly, 3 among the 5 changes were at Rest 2 which is only 15 min after treatment and might possibly indicate faster, though limited, effects following direct-entry. A recent regional cerebral blood flow study has also reported some neural effects of intranasal OXT after around 15-23 mins [27]. The limited effects of direct-entry of OXT might also be helpful in explaining some of the cases whereby delta-beta_low_ CFC following PLC + OXT was only significantly different from either following VC + OXT or VC + PLC treatments but not both. Other factors such as statistical variations caused by individual differences of temporal metabolism of OXT, endogenous OXT levels, or oxytocinergic responsivity [59, 60] can also contribute to these inconsistencies. However, robust treatment effects were consistently found in Rest 4 (45 min post-treatment) for both the delta-beta_low_ and beta_high_ CFC and less robust effects at other timepoints may have been more influenced by such individual differences.

Additional support for exogenously administered OXT producing functional effects via increasing its peripheral concentrations comes from studies where it is given via routes which preclude direct-entry into the brain (i.e., intravenous, intraperitoneal, oral and subcutaneous) [23]. Notably in rodent models of autism, intraperitoneal and subcutaneous routes of OXT administration have often been used to successfully rescue social deficits [61, 62]. In humans, intravenously administered OXT was initially reported to improve social symptoms in autistic adults [25, 26]. Most recently in humans we have shown that oral OXT increases brain reward and amygdala as well as arousal responses to emotional faces, with enhanced responses in the putamen to happy faces being correlated with increased blood concentrations [28]. Similarly, we have shown that oral and intranasal OXT produce indistinguishable effects on social attention and anxiety [29, 30], with behavioral effects also associated with increased blood concentrations [29].

So what mechanism(s) may be involved in the ability of OXT to produce effects on brain and behavior via increases in peripheral concentrations? The first mechanism could be by increased peripheral concentrations of OXT crossing the BBB to enter the brain. Even though early studies found that the BBB is relatively impermeable to OXT [15, 16], several recent animal studies using labeled OXT have reported that it can enter the brain after intravenous administration [63, 64]. The discovery in rodents that OXT can enter the brain from the peripheral circulation after binding to RAGE has provided a possible mechanism. It has been reported that functional effects of both intranasal and subcutaneous OXT do not occur in RAGE knockout mice and increased brain concentrations of OXT are prevented [65–67]. In vitro studies have also reported the presence of this potential transport system in humans [68]. Another potential mechanism is that increased peripheral OXT concentrations act on its receptors in the heart and gastrointestinal system to promote vagally-mediated stimulation of the brain [23, 69, 70]. Given some previous evidence for intranasal OXT influencing parasympathetic/vagal activity in terms of HRV [36, 71], SCR [43, 44, 72] and gastrointestinal mobility [47], we had anticipated that we would find some support for this but in line with some other reports we did not observe any significant effects on ECG [27, 45, 46], SCR, or EGG parameters. While this does not rule out possible vagally-mediated effects we were unable in the current study to demonstrate any evidence for peripheral vagal stimulation by OXT. At this stage therefore, while it is unclear what specific mechanism(s) are involved in peripherally-mediated effects of OXT on the brain, the absence of evidence for vagal stimulation could indicate that entry into the brain via the BBB is the most likely.

The present study has the following limitations. First, VC + PLC treatment was used as the only control condition and thus it was not a fully balanced design due to a lack of a PLC + PLC treatment condition. However, given that plasma OXT concentrations remained stable at a very low level over timepoints following the VC + PLC treatment, and that subjects were unable to identify better than chance whether they had received VC or PLC, we believe that the VC + PLC treatment represents a sufficient control condition. Second, only males were recruited in the present study and thus the findings cannot be extended to females. Third, the methodologies used in the present study did not allow us to distinguish specific contributions by the centrally- and peripherally-mediated effects of intranasal OXT. Future studies are needed to additionally determine whether OXT administered via a purely peripheral route can induce similar effects on delta-beta coupling to the present study after determining the exact doses of peripheral administration that can achieve similar plasma OXT concentrations to intranasal administration. This is important given some evidence for dose-dependent effects of intranasal OXT [27, 73–75].

In conclusion, we provide the first evidence for the efficacy of a novel treatment approach, combining VC pretreatment with intranasal OXT administration, in effectively decreasing intranasally applied OXT entering the peripheral circulation. Importantly, this resulted in greatly reducing increased delta-beta CFC induced by conventional intranasal administration of OXT, with only a very few regions still exhibiting delta-beta CFC changes following VC pretreatment. These findings suggest a critical role of peripheral vasculature-mediated routes in functional actions of intranasal OXT and since we did not find any modulatory effects of intranasal OXT on parasympathetic/vagal physiological responses of peripheral system it is possible that effects on brain function may have resulted from OXT crossing the BBB. Our findings are of great potential therapeutic significance both in terms of routes whereby OXT can be administered during interventions in psychiatric disorders and in terms of the utility of VC pretreatment for minimizing entry of drugs administered intranasally into the peripheral circulation which might result in adverse effects.

### Supplementary information

Supplemental Materials

## Data Availability

The data that support the findings of this study are available from the corresponding author on reasonable request and with permission of university administration.
