# Supplementary material for: Sniffing oxytocin: Nose to brain or nose to blood?
Source: Mol Psychiatry. 2023 Apr 25;28(7):3083–91. doi: 10.1038/s41380-023-02075-2 (PMC10615745; doi:10.1038/s41380-023-02075-2)
Supplement: Supplementary file 1 — Supplemental Materials [file 41380_2023_2075_MOESM1_ESM.pdf]

Supplementary Information

Questionnaires

To control potential confounding effects from mood states or personality traits, subjects completed Chinese versions of validated questionnaires before treatment, including the Positive and Negative Affect Schedule (PANAS) [1], State-Trait Anxiety Inventory [2], Beck Depression Inventory-II [3], Autism Spectrum Quotient [4], Interpersonal Reactivity Index [5]. Subjects completed the PANAS when they arrived the lab (pre-test) and at the end of the experiment (post-test).

Data Acquisition and Analyses

Plasma OXT concentration analyses

Blood samples for OXT assay were collected in 6 ml EDTA tubes and centrifuged at 1600 g for 15 min at 4 °C within half an hour after collection. Plasma was then aliquoted into chilled safe-lock tubes (Sarstedt, Germany) and stored at -80 °C for subsequent OXT concentration analyses. All samples were analyzed within 6 months after collection. OXT concentrations in 1 ml plasma samples were measured using a 96-plate commercial ELISA assay kit (ENZO Life Science, Farmingdale, NY, USA, Catalog #: ADI-901-153A). The manufacturer quotes a sensitivity of 15 pg/ml with no concentration of samples but does not provide intra-or inter-assay coefficients of variation. Following recommendations from the manufacturer, a standard prior extraction step was employed and spiked samples (with 100 pg/ml OXT added) were included in every assay for extraction efficiency calculation (96.6%). The extraction procedure incorporated a 4-fold concentration of samples using a vacuum

concentrator (Concentrator plus, Eppendorf, Germany) and based on our standard curves from 27 plates we achieved a detection sensitivity of 2.5 pg/ml (i.e. 10 pg/ml without the concentration factor) using the manufacturers recommendation for sensitivity calculations. Where sample concentrations were below the detection sensitivity they were assigned the average detection sensitivity of 2.5 pg/ml (this only occurred for < 0.5% of samples). All measurements were performed in duplicate and the concentrations were calculated using a multimode plate reader (Tecan Infinite M200 PRO, Austria) at 405 nm according to standard curves. Intra- and inter-assay coefficients of variation were 9.7% and 6.9% respectively. Performance of the assay and calculated plasma OXT concentrations are similar to those we have reported in a number of previous studies [6-9]. To minimize influences of individual differences of baseline plasma OXT concentrations [10, 11], ratios of plasma OXT concentrations were calculated to feature pharmacokinetic profiles of plasma OXT over time by dividing raw plasma OXT concentrations at each timepoint by raw values at baseline. To obtain a more stable measurement of OXT concentrations at baseline, baseline values were an average of the two blood samples taken before treatment.

Resting State EEG Recording and Data Processing

The rsEEG was recorded at a sampling rate of 500 Hz using a 64-channel actiCHamp system (Brain Products GmbH, Germany), with on-line reference on the Cz electrode and the ground on a medial prefrontal electrode. Electrode impedance was kept below 10 kΩ. Offline EEG data were preprocessed using the EEGLAB v2019.0 toolbox [12]. The raw EEG data were down sampled to 250 Hz and filtered with a 0.5-45 Hz

band-pass using a windowed sinc FIR filter via the ‘pop_eegfiltnew’ function of the EEGLAB toolbox. Bad channels were identified using the EEGLAB clean_rawdata plugin and were interpolated using the spherical interpolation method. After re-referencing the EEG data to the average reference, an independent component analysis was performed and the ICLabel plugin was used to reject components related to eye movements and muscle activity [13]. The resultant EEG data was then segmented into non-overlapping 2 s epochs (150 trials) and epochs with voltage values exceeding ± 80 μv were deleted. A mean percentage of 17.38% trials (range from 15.99%-18.96%) were excluded for each rsEEG data.

Cleaned EEG epochs were exported for Fast Fourier Transform. Averaged spectral power values were then calculated for delta (1-4 Hz), low beta ($beta_{low}$, 13-25 Hz), and high beta ($beta_{high}$, 25-35 Hz) frequencies respectively. Power values were further averaged across electrodes for prefrontal (Fp1, Fp2), frontal (F3, F4, Fz), central (C3, C4, Cz), parietal (P3, P4, Pz), temporal (T7, T8), and occipital (O1, O2, Oz) regions based on the 10-20 electrode system (see Supplementary Figure S1). Power-based connectivity was then computed between each pair of the 6 regions (inter-regional CFC) and within each region (intra-regional CFC) following guidelines in Cohen (2014) [14]. This led to 6 × 5 = 30 pairs of inter-regional delta-$beta_{low}$/$beta_{high}$ CFC and 6 pairs of intra-regional and delta-$beta_{low}$/$beta_{high}$ CFC. More specifically, correlation coefficients between power time series of the delta and beta bands were firstly computed on each trial and then averaged across trials to increase the signal-to-noise ratio. Correlation coefficients were calculated using the Spearman

correlation method and were then Fisher-Z transformed for subsequent statistical analyses [14].

Phase-amplitude coupling (PAC) was also analyzed based on these cleaned EEG epochs by applying the traditional method (Mean Vector Length-MVL) introduced by Canolty et al. (2006) [15]. Time series of the filtered delta and $beta_{high}$/$beta_{low}$ frequencies were first averaged across electrodes for prefrontal (Fp1, Fp2), frontal (F3, F4, Fz), central (C3, C4, Cz), parietal (P3, P4, Pz), temporal (T7, T8), and occipital (O1, O2, Oz) regions based on the 10-20 electrode system respectively (see Figure S1). Delta phase angle and $beta_{high}$/$beta_{low}$ magnitude were then extracted from the Hilbert-transformed data of the delta and $beta_{high}$/$beta_{low}$ signal. To remove edge artefacts derived from filtering, data of the first and last 8 data points were deleted from each epoch. The observed delta-$beta_{high}$/$beta_{low}$ PAC was then calculated based on delta phase angle and $beta_{high}$/$beta_{low}$ magnitude of each data point using the following formula:

$$PAC = \left| \frac{\sum_{t=1}^{n} a_t e^{i\theta_t}}{n} \right|$$

Where n is the total number of data points, t is a specific data point, a_t is the amplitude of $beta_{high}$/$beta_{low}$ at data point t, and θ_t is the phase angle of delta at data point t. A permutation testing procudure was then employed for 1000 times and shuffled PAC ($PAC_{shuffled}$) values were calculated between the time series of the delta phase and a permuted $beta_{high}$/$beta_{low}$ amplitude time series, which was constructed by randomly shuffling the amplitude time series. The observed PAC values were standardized to the distribution of the shuffled PAC values using the following formula:

$$PAC_z = \frac{PAC - mean(PAC_{\text{shuffled}})}{std(PAC_{\text{shuffled}})}$$

These PAC Z-values were computed between each pair of the 6 regions (inter-regional PAC) and within each region (intra-regional PAC) for delta-beta$_{\text{high}}$ PAC and delta-beta$_{\text{low}}$ of each subject. These Z values were used for subsequent hypothesis tests given their straightforward interpretation [14, 16].

Physiological Data Recording and Processing

Physiological measurements were recorded at a sampling rate of 1000 Hz using a BIOPAC MP150 system (BIOPAC Systems, Inc.) in accordance with the BIOPAC manual. Skin conductance response (SCR) was recorded using a GSR100C module with two electrodes placed on the participant's fingertips. ECG was recorded using an ECG100C module with two electrodes placed on the lower right leg and the right arm. For electrogastrogram (EGG), the EGG100C module was used and two electrodes were placed below the costal margin (approximately 6 cm left of the midline) and approximately 3 cm above the navel (approximately 3 cm left of the midline). The ground electrode was placed on the right lower limb.

Physiological data was processed using AcqKnowledge v4.4 software following recommendations of the manual or previous studies. In line with previous studies [17, 18], the raw SCR data was first down-sampled to 62.5 Hz followed by a median filter smooth. A 1 Hz low-pass filter was then conducted to determine the tonic EDA component. The resultant data was log-transformed and the averaged SCR was extracted for subsequent statistical analyses. The ECG data was manually inspected prior to analyses and quality of most of the data was high. Next, the data was band

pass filtered (0.5-35 Hz) to remove baseline drift and high frequency noise. Data still with artifacts were manually corrected using the Waveform Math function. R-R intervals were then extracted from the clean ECG and imported to Kubios software (available at: http://kubios.uku.fi) for HRV analyses. Consistent with previous studies examining OXT effects on HRV [19, 20], mean heart rate (HR), high frequency HRV (HF-HRV), the detrended fluctuation scaling exponent (DFAα1) were extracted to test for treatment group differences. Logarithmic transformation was applied where variables were not normally distributed. For EGG, consistent with a previous study [21], data were low-pass filtered below 5 Hz to avoid aliasing. The filtered data were then manually inspected and mean amplitude and cycles per min (CPM) were exported for subsequent analyses.

Results of plasma OXT concentration changes in ratios

Plasma OXT concentration changes in ratios relative to baseline over time are presented in Figure S2. Plasma OXT concentrations reached a peak at 15 min post-treatment in samples collected in the present study. A repeated-measures ANOVA was performed on ratios of plasma OXT concentrations following treatment and showed significant main effects of timepoint ($F(3.52, 309.47) = 19.51$, $p < 0.001$, $\eta_p^2 = 0.181$) and treatment ($F(2, 88) = 19.01$, $p < 0.001$, $\eta_p^2 = 0.302$). Importantly, the interaction between timepoint and treatment was significant ($F(7.03, 309.47) = 7.72$, $p < 0.001$, $\eta_p^2 = 0.149$). Post-hoc analyses revealed significantly higher OXT concentrations from 15 to 60 min following PLC + OXT in comparison to VC + OXT (15 min: $p < 0.001$; 30 min: $p < 0.001$; 45 min: $p = 0.003$; 60 min: $p = 0.016$) and VC + PLC

treatments (15 min: $p < 0.001$; 30 min: $p < 0.001$; 45 min: $p < 0.001$; 60 min: $p = 0.002$). There were no siginificant differences over time between VC + OXT and VC + PLC treatments ($ps \geq 0.123$).

Results of delta and beta$_{low}$/beta$_{high}$ power

Repeated-measures ANOVAs were conducted on delta, beta$_{low}$, and beta$_{high}$ power values with timepoint and location (prefrontal, frontal, central, parietal, temporal, and occipital) as within-subject factor and treatment as between-subject factor. For delta band, there was a significant main effect of timepoint ($F(4.35, 382.86) = 11.05$, $p < 0.001$, $\eta_p^2 = 0.112$) and location ($F(3.26, 286.55) = 50.10$, $p < 0.001$, $\eta_p^2 = 0.363$). The interaction between timepoint and location was also significant ($F(12.38, 1088.99) = 1.77$, $p = 0.047$, $\eta_p^2 = 0.020$). However, there were no significant effects related to treatment or other significant effects (all $ps \geq 0.125$). For beta$_{low}$ band and beta$_{high}$, the main effect of timepoint (beta$_{low}$: $F(4.08, 359.03) = 9.93$, $p < 0.001$, $\eta_p^2 = 0.101$; beta$_{high}$: $F(4.60, 404.98) = 8.61$, $p < 0.001$, $\eta_p^2 = 0.089$) and location (beta$_{low}$: $F(2.36, 207.51) = 42.89$, $p < 0.001$, $\eta_p^2 = 0.328$; beta$_{high}$: $F(4.05, 355.99) = 26.58$, $p < 0.001$, $\eta_p^2 = 0.232$) were significant. Similarly, there were also no significant effects related to treatment or other significant effects (all $ps \geq 0.171$ for beta$_{low}$ and all $ps \geq 0.257$ for beta$_{high}$).

Results of individual delta-beta$_{low}$ CFC

For each inter- and intra-regional delta-beta$_{low}$ CFC, significant interaction effects between timepoint and treatment were found in 28 among 30 pairs of inter-regional ($ps \leq 0.024$, FDR corrected) and in 4 among 6 pairs of intra-regional delta-beta$_{low}$

CFC (ps ≤ 0.032, FDR corrected; see Supplementary Figure S3A for p values of each pair). Post hoc analyses revealed a generally similar pattern of individual regional CFCs across treatments over timepoints to the global one (see Supplementary Figure S4). Note that although we did not find evidence for a direct entry-driven effect (i.e., effects of VC + OXT treatment) at a global level, at an individual regional level there were similar enhancement effects of PLC+ OXT and VC + OXT treatments on central delta-parietal $beta_{low}$ (PLC + OXT: $p = 0.017$; VC + OXT: $p = 0.014$), parietal delta-central $beta_{low}$ (PLC + OXT: $p = 0.005$; VC + OXT: $p = 0.087$), and central delta-temporal $beta_{low}$ (PLC + OXT: $p = 0.026$; VC + OXT: $p = 0.099$) CFCs in Rest 3 compared to VC + PLC treatment, although the effect of VC + OXT was only significant for central delta-parietal $beta_{low}$. VC + OXT treatment also significantly increased temporal delta-frontal $beta_{low}$ CFC ($p = 0.041$) and temporal delta-occipital $beta_{low}$ CFC relative to VC + PLC treatment in Rest 2 ($p = 0.025$; Supplementary Figure S4).

Results of individual delta-$beta_{high}$ CFC

For each inter- and intra-regional delta-$beta_{high}$ CFC, significant interaction effects between timepoint and treatment were found in 13 among 30 pairs of inter-regional (ps ≤ 0.048, FDR corrected) and in 2 among 6 pairs of intra-regional delta-$beta_{high}$ CFC (ps = 0.032, FDR corrected; see Supplementary Figure S3B for p values of each pair). Post hoc analyses revealed a generally similar pattern of individual CFC across treatments over timepoints to the global one (see Supplementary Figure S5). For delta-$beta_{high}$ CFC, we also found evidence for a direct entry-driven effect in that PLC

+ OXT and VC + OXT treatments showed similar enhancement effects on increasing prefrontal delta-frontal $beta_{high}$ CFC in Rest 5 (PLC + OXT: $p = 0.087$; VC + OXT: $p = 0.040$). VC + OXT treatment was also found to increase central delta-frontal $beta_{high}$ CFC relative to VC + PLC treatment in Rest 2 ($p = 0.002$; Supplementary Figure S5).

Results of phase-amplitude coupling

Repeated-measures ANOVAs were conducted on Z values of PAC with timepoint (Rest 1-7) as within-subject factor and treatment as between-subject factor. The main effect of both the timepoint (global: $F(4.89, 430.30) = 0.70$, $p = 0.620$, $\eta_p^2 = 0.008$; inter-regional: $F(4.88, 429.74) = 0.69$, $p = 0.628$, $\eta_p^2 = 0.008$; intra-regional: $F(4.95, 435.67) = 0.72$, $p = 0.610$, $\eta_p^2 = 0.008$) and treatment (global: $F(2, 88) = 1.88$, $p = 0.159$, $\eta_p^2 = 0.041$; inter-regional: $F(2, 88) = 1.84$, $p = 0.164$, $\eta_p^2 = 0.040$; intra-regional: $F(2, 88) = 1.94$, $p = 0.149$, $\eta_p^2 = 0.042$) were not significant for all of the global, inter-regional and intra-regional delta-$beta_{low}$ PAC. The interaction between timepoint and treatment was also not significant (global: $F(9.78, 430.30) = 0.84$, $p = 0.590$, $\eta_p^2 = 0.019$; inter-regional: $F(9.78, 429.74) = 0.89$, $p = 0.545$, $\eta_p^2 = 0.020$; intra-regional: $F(9.90, 435.67) = 0.66$, $p = 0.764$, $\eta_p^2 = 0.015$).

Similar to delta-$beta_{low}$ PAC, none of the main effect of timepoint (global: $F(5.15, 452.99) = 0.54$, $p = 0.753$, $\eta_p^2 = 0.006$; inter-regional: $F(5.19, 456.87) = 0.49$, $p = 0.792$, $\eta_p^2 = 0.006$; intra-regional: $F(5.08, 447.35) = 0.72$, $p = 0.612$, $\eta_p^2 = 0.008$) and treatment (global: $F(2, 88) = 0.37$, $p = 0.690$, $\eta_p^2 = 0.008$; inter-regional: $F(2, 88) = 0.34$, $p = 0.711$, $\eta_p^2 = 0.008$; intra-regional: $F(2, 88) = 0.53$, $p = 0.589$, $\eta_p^2 = 0.012$) was significant for all of the global, inter-regional and intra-regional delta-$beta_{high}$

PAC. The interaction between timepoint and treatment was also not significant (global: $F(10.30, 452.99) = 0.71$, $p = 0.724$, $\eta_p^2 = 0.016$; inter-regional: $F(10.38, 456.87) = 0.73$, $p = 0.707$, $\eta_p^2 = 0.016$; intra-regional: $F(10.17, 447.35) = 0.61$, $p = 0.809$, $\eta_p^2 = 0.014$).

SCR results

A repeated-measures ANOVA was performed on averaged SCR values with timepoint as within-subject factor and treatment as between-subject factor. This revealed a significant main effect of timepoint ($F(6, 528) = 5.29$, $p = 0.014$, $\eta_p^2 = 0.057$), with a higher SCR at Rest 2 than the followed timepoints from Rest 3 to 7 (all ps ≤ 0.004; Supplementary Figure S6). However, the main effect of treatment ($F(2, 88) = 0.50$, $p = 0.609$, $\eta_p^2 = 0.011$) and interaction between timepoint and treatment ($F(12, 528) = 0.87$, $p = 0.451$, $\eta_p^2 = 0.019$) were not significant.

ECG results

Repeated-measures ANOVAs revealed significant main effects of timepoint for all the three HRV indices (mean HR: $F(3.90, 343.16) = 5.98$, $p < 0.001$, $\eta_p^2 = 0.064$; HF-HRV: $F(4.11, 361.31) = 3.42$, $p = 0.009$, $\eta_p^2 = 0.037$; DFAα1: $F(5.14, 451.95) = 8.79$, $p < 0.001$, $\eta_p^2 = 0.091$), with the mean HR and HF-HRV reaching the peak in Rest 2 and 3 respectively, followed by a decreasing trend in the remaining timepoints (Supplementary Figure S7A and S7B). The DFAα1 showed a general increasing trend from Rest 1 to 7 (all ps ≤ 0.012; Supplementary Figure S7C). There were no other significant main effects or interactions (all ps ≥ 0.127).

EGG results

Similar to other physiological indices, the repeated-measures ANOVA on mean amplitude of EGG revealed a significant main effect of timepoint ($F(2.24, 196.72) = 5.61$, $p = 0.003$, $\eta_p^2 = 0.060$), with the mean amplitude in Rest 1 being significantly higher than Rest 7 ($p = 0.032$) (Supplementary Figure S8A). For CPM, the main effect of timepoint was also significant ($F(4.46, 392.09) = 12.97$, $p < 0.001$, $\eta_p^2 = 0.128$), with a general decreasing trend from Rest 1 to 7 (Supplementary Figure S8B). There were no other significant main effects or interaction (all ps ≥ 0.187).

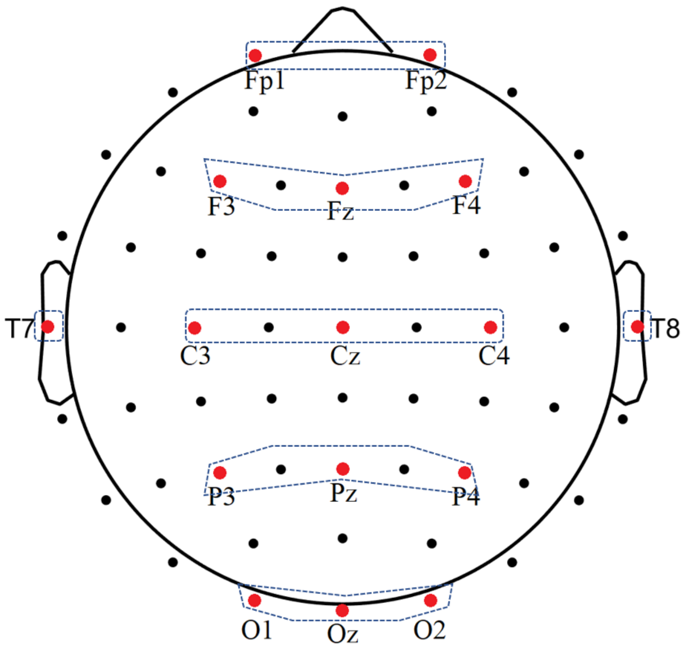

Figure S1. Illustration of electrodes for prefrontal (Fp1, Fp2), frontal (F3, F4, Fz), central (C3, C4, Cz), parietal (P3, P4, Pz), temporal (T7, T8), and occipital (O1, O2, Oz) regions based on the 10-20 electrode system.

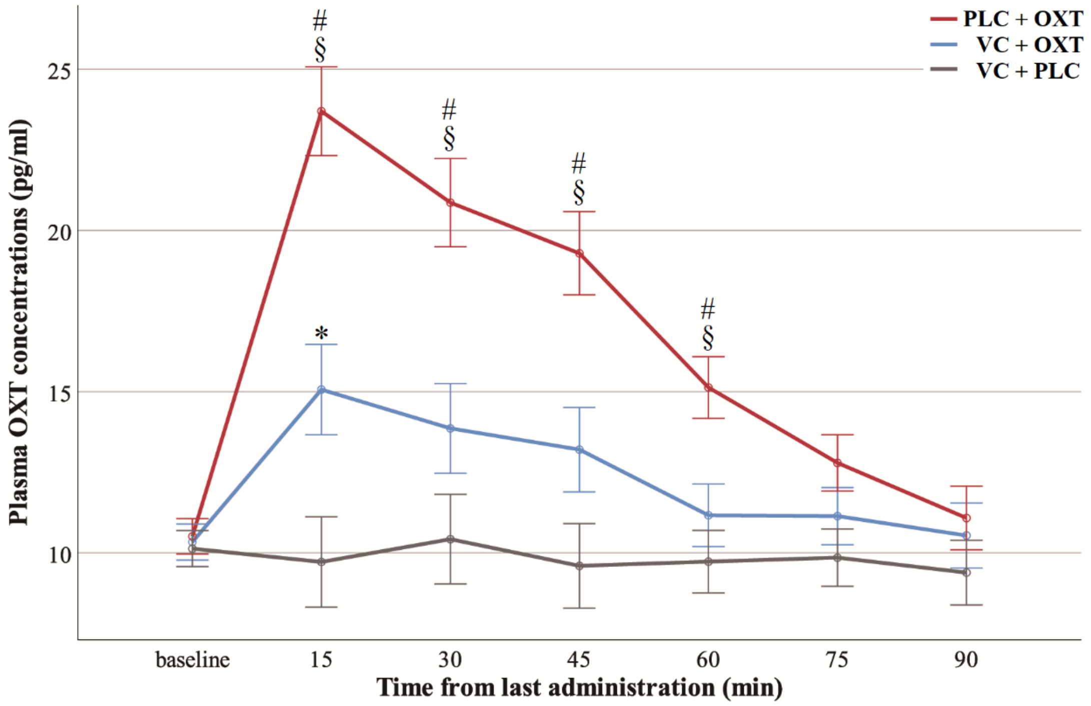

Figure S2. Pharmacokinetic profiles of plasma oxytocin (OXT - ratio relative to baseline) over time for each treatment. Baseline is an average of blood sample 1 and 2 and serves as baseline. The remaining timepoints (15-90 min) correspond to blood samples 3-8. Data presented are mean ± standard error. "§" indicates significantly higher OXT concentrations following PLC + OXT in comparison to VC + PLC ($p < 0.05$ FWE corrected). "#" indicates significantly higher OXT concentrations following PLC + OXT in comparison to VC + OXT ($p < 0.05$ FWE

corrected). OXT: oxytocin. VC: vasoconstrictor. PLC: placebo.

A

	PF $beta_{low}$	Fron $beta_{low}$	Cen $beta_{low}$	Tem $beta_{low}$	Par $beta_{low}$	Occ $beta_{low}$
PF delta	0.091	0.021	0.021	0	0.014	0.005
Fron delta	0.009	0.054	0.14	0.005	0.002	0.004
Cen delta	0.009	0	0.032	0.004	0.002	0
Tem delta	0.012	0.002	0.024	0.006	0.002	0.002
Par delta	0.013	0.002	0.008	0.002	0	0.002
Occ delta	0.011	0.002	0.129	0	0.007	0.032

0 — 0.14

B

	PF $beta_{high}$	Fron $beta_{high}$	Cen $beta_{high}$	Tem $beta_{high}$	Par $beta_{high}$	Occ $beta_{high}$
PF delta	0.732	0.048	0.414	0.194	0.005	0.003
Fron delta	0.465	0.844	0.08	0.468	0.146	0.089
Cen delta	0.033	0	0.599	0.615	0.026	0
Tem delta	0.003	0	0.257	0.032	0	0
Par delta	0.226	0.502	0.321	0.187	0.032	0.048
Occ delta	0.064	0.08	0.179	0.329	0.023	0.053

0 — 0.844

Figure S3. P value matrixes of interaction effects between timepoint and treatment for delta-$beta_{low}$ (A) and delta-$beta_{high}$ CFCs (B). All p values reported are FDR corrected.

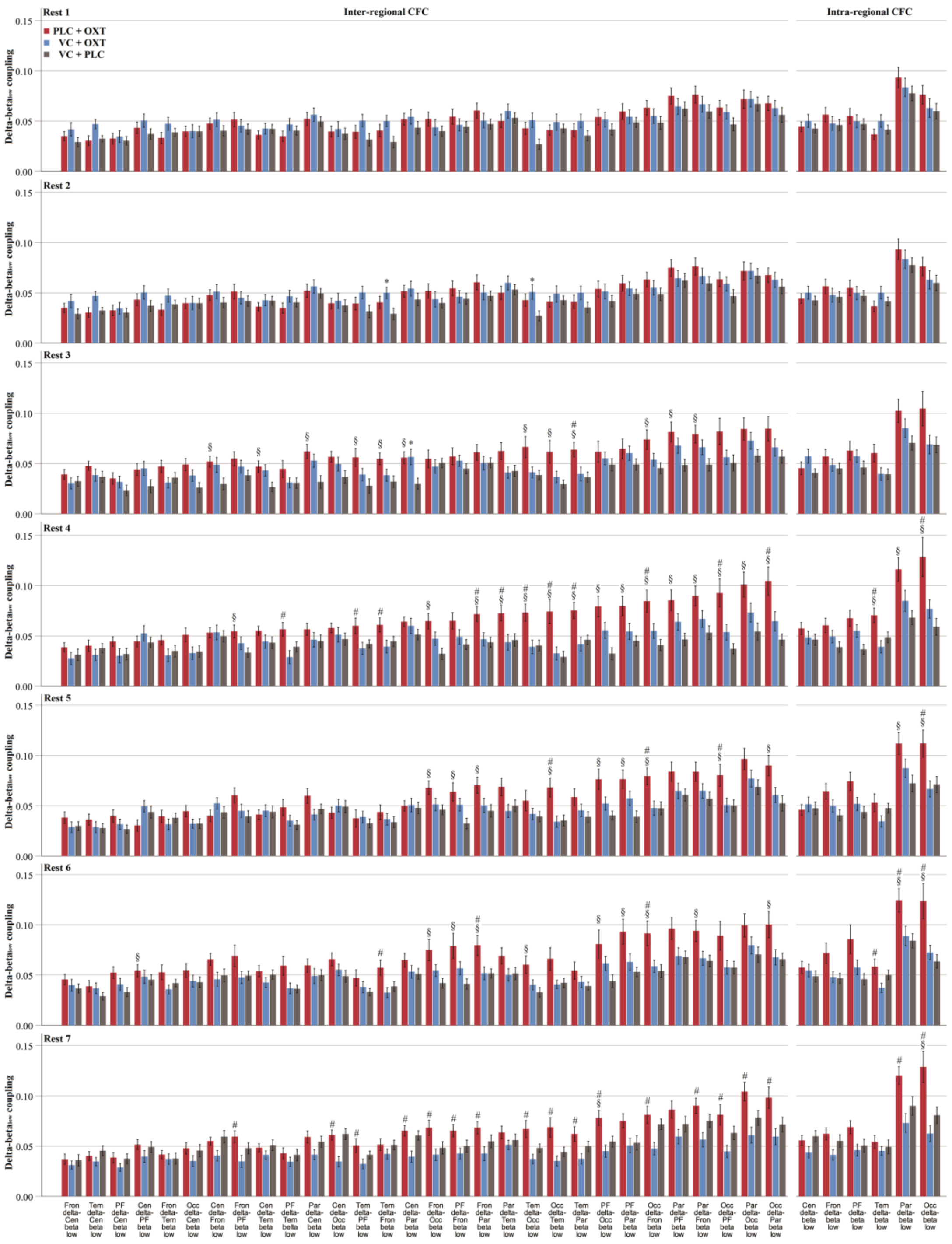

Figure S4. Patterns of individual regional delta-beta$_{low}$ CFCs for each treatment over timepoints (Rest 1-7). "§" indicates significantly stronger delta-beta$_{low}$ CFCs following PLC + OXT than VC + PLC ($p < 0.05$ FWE corrected). "#" indicates significantly stronger delta-beta$_{low}$ CFCs following PLC + OT than VC + OXT ($p < 0.05$ FWE corrected). "*" indicates significantly stronger delta-beta$_{low}$ CFCs following VC + OT than VC + PLC ($p < 0.05$ FWE corrected). Data presented are mean ± standard error.

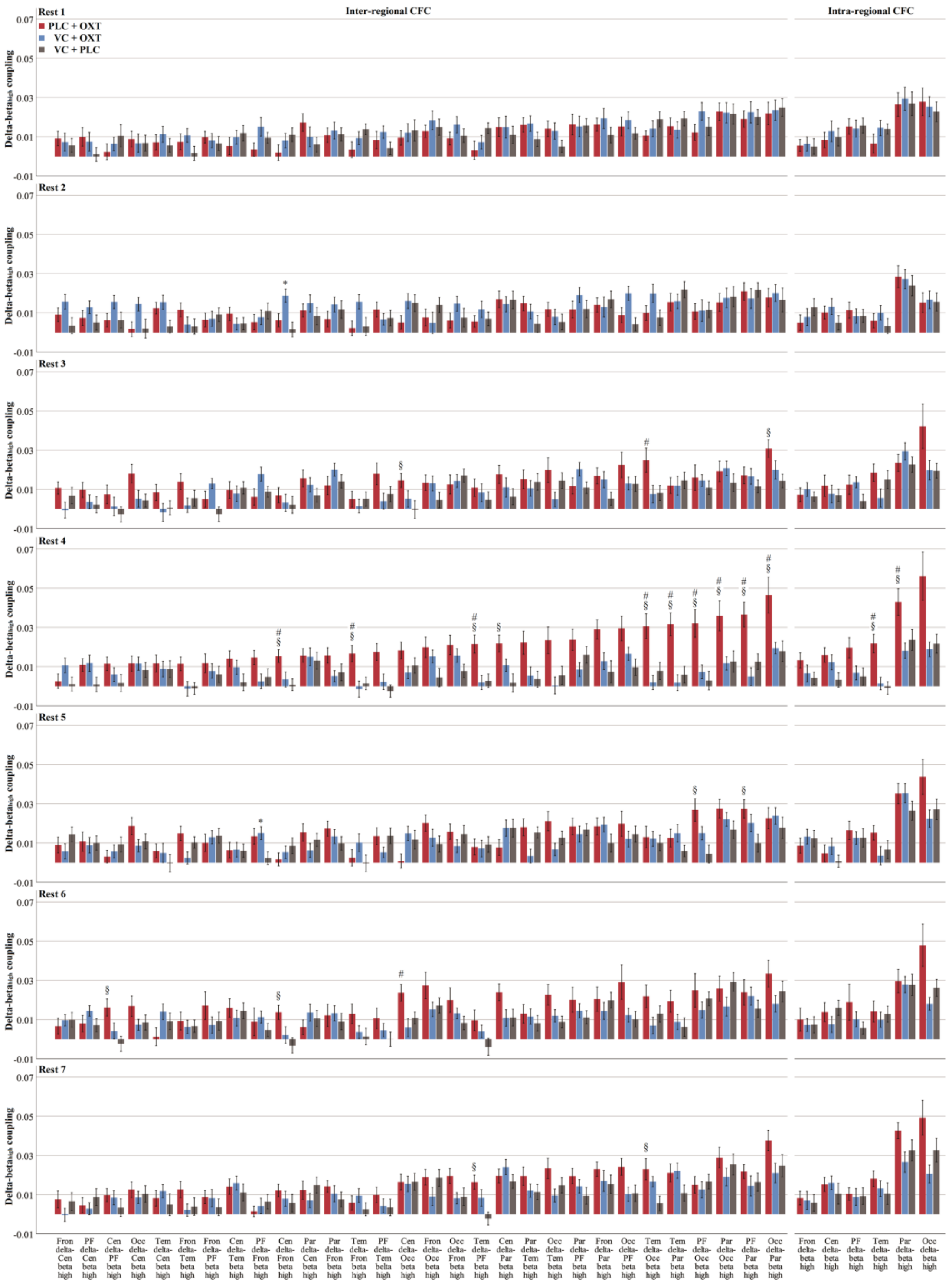

Figure S5. Patterns of individual regional delta-beta$_{high}$ CFCs for each treatment over timepoints (Rest 1-7). "§" indicates significantly stronger delta-beta$_{high}$ CFCs following PLC + OXT than VC + PLC ($p < 0.05$ FWE corrected). "#" indicates significantly stronger delta-beta$_{high}$ CFCs following PLC + OT than VC + OXT ($p < 0.05$ FWE corrected). "*" indicates significantly stronger delta-beta$_{high}$ CFCs following VC + OT than VC + PLC ($p < 0.05$ FWE corrected). Data presented are mean ± standard error.

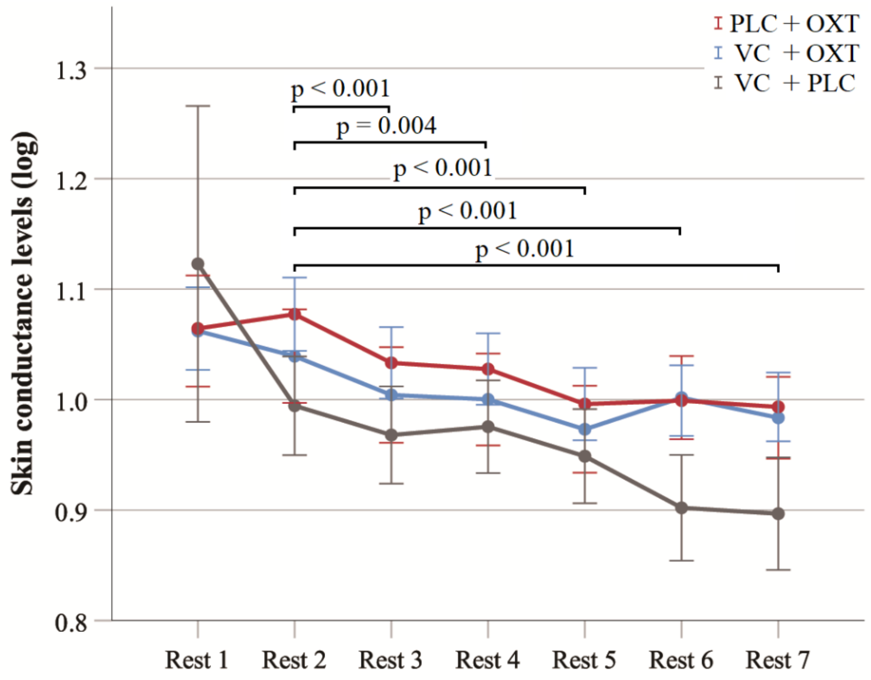

Figure S6. Patterns of skin conductance levels for each treatment from Rest 1 to 7. Data presented are mean ± standard error.

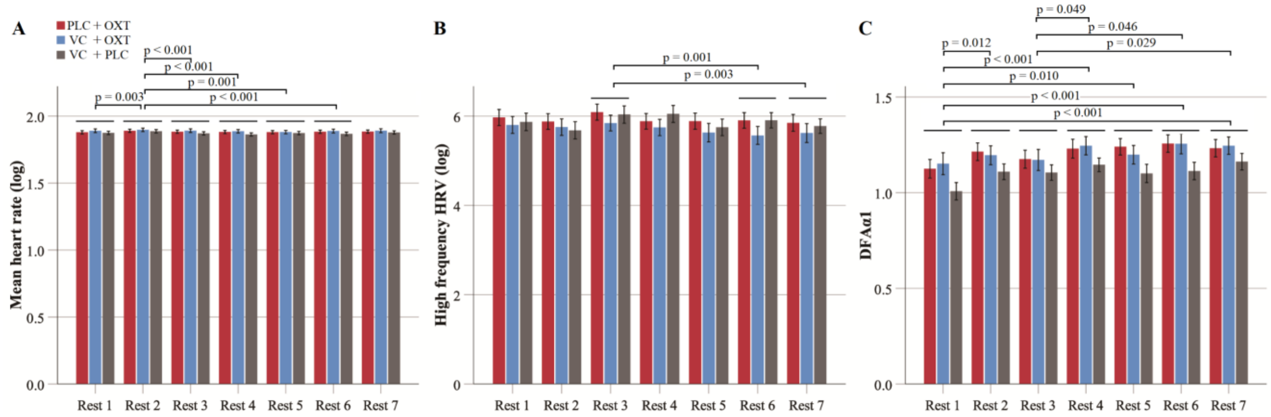

Figure S7. Patterns of heart rate (A), high frequency heart rate variability (HRV) (B), and the detrended fluctuation scaling exponent (DFAα1) (C) for each treatment from Rest 1 to 7. Data presented are mean ± standard error.

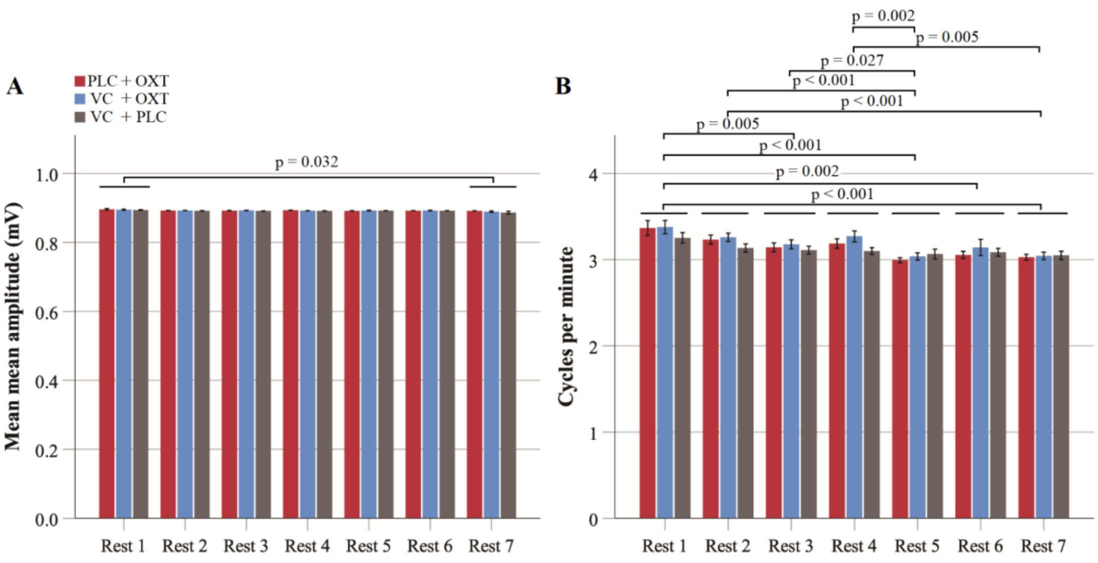

Figure S8. Patterns of the mean amplitde (A) and cycles per minute (B) of the electrogastrogram for each treatment from Rest 1 to 7. Data presented are mean ± standard error.

Table S1. Statistics of questionnaire scores for each treatment (Mean ± SD).

Measurements	VC+OXT	PLC+OXT	VC+PLC	F	*p*
Age	21.0±1.8	21.6±1.8	21.3±2.1	0.64	0.530
PANAS-pre					
- Positive	26.4±5.7	25.5±6.3	24.4±6.2	0.76	0.470
- Negative	14.0±5.0	12.1±3.8	13.3±3.7	1.62	0.203
PANAS-post					
- Positive	22.0±7.7	21.5±7.1	21.2±5.6	0.12	0.886
- Negative	11.7±4.5	11.2±3.1	12.1±3.4	0.52	0.598
State-Trait Anxiety Inventory					
- State	38.0±8.0	35.0±4.9	38.6±9.0	2.08	0.131
- Trait	40.5±6.8	38.1±6.2	39.7±8.6	0.89	0.414
Beck Depression Inventory	7.6±5.8	7.7±6.3	6.8±5.6	0.19	0.831
Autism Spectrum Quotient	22.9±6.3	22.0±5.3	21.1±5.7	0.70	0.501
Interpersonal Reactivity Index	50.8±8.2	49.1±10.3	47.4±10.9	0.91	0.408

95% Confidence interval. PANAS: Positive and Negative Affect Schedule.

Table S2. Statistics for repeated-measures ANOVAs on Fisher-Z transformed correlation coefficients.

Cross-frequency couplings	Phase			Treatment			Phase*Treatment		
	F	P	η_p^2	F	P	η_p^2	F	P	η_p^2
Global delta-beta$_{low}$	2.66	0.021	0.029	3.46	0.036	0.073	3.73	0.000	0.078
Inter-regional delta-beta$_{low}$	2.50	0.030	0.028	3.24	0.044	0.069	3.74	0.000	0.078
Intra-regional delta-beta$_{low}$	3.34	0.005	0.037	4.43	0.015	0.092	3.46	0.000	0.073
Global delta-beta$_{high}$	0.59	0.712	0.007	2.93	0.059	0.062	2.43	0.008	0.052
Inter-regional delta-beta$_{high}$	0.43	0.831	0.005	2.81	0.066	0.060	2.33	0.011	0.050
Intra-regional delta-beta$_{high}$	1.65	0.144	0.018	3.28	0.042	0.069	2.30	0.011	0.050

References

1. Watson D, Clark LA, Tellegen A. Development and validation of brief measures of positive and negative affect: the PANAS scales. *J Pers Soc Psychol* 1988; **54**: 1063.
2. Spielberger CD, Gorsuch R, Lushene R, Vagg PR, Jacobs GA. *Manual for the state-trait anxiety inventory*. Consulting Psychologists Press: Palo Alto, 1983.
3. Beck AT, Steer RA, Ball R, Ranieri WF. Comparison of Beck Depression Inventories-IA and-II in psychiatric outpatients. *J Pers Assess* 1996; **67**: 588-597.
4. Baron-Cohen S, Wheelwright S, Skinner R, Martin J, Clubley E. The autism-spectrum quotient (AQ): Evidence from asperger syndrome/high-functioning autism, males and females, scientists and mathematicians. *J Autism Dev Disord* 2001; **31**: 5-17.
5. Davis MH. Measuring individual differences in empathy: Evidence for a multidimensional approach. *J Pers Soc Psychol* 1983; **44**: 113.
6. Chen Y, Li Q, Zhang Q, Kou J, Zhang Y, Cui H et al. The effects of intranasal oxytocin on neural and behavioral responses to social touch in the form of massage. *Front Neurosci* 2020; **14**: 589878.
7. Kou J, Lan C, Zhang Y, Wang Q, Zhou F, Zhao Z et al. In the nose or on the tongue? Contrasting motivational effects of oral and intranasal oxytocin on arousal and reward during social processing. *Transl Psychiatry* 2021; **11**: 94.
8. Le J, Zhang L, Zhao W, Zhu S, Lan C, Kou J et al. Infrequent intranasal oxytocin followed by positive social interaction improves symptoms in autistic children: a

pilot randomized clinical trial. *Psychother Psychosom* 2022; **91**: 335-347.

9. Xu D, Li Q, Zhuang Q, Zhang Y, Yao S, Zhao W et al. Oro-mucosal administration of oxytocin using medicated lollipops alters social attention, similar to intranasal and lingual routes: Implications for therapeutic use. *Front Neurosci* 2022; **16**: 1022101.
10. Bartz JA, Zaki J, Bolger N, Ochsner KN. Social effects of oxytocin in humans: context and person matter. *Trends Cogn Sci* 2011; **15**: 301-309.
11. Crockford C, Deschner T, Ziegler TE, Wittig RM. Endogenous peripheral oxytocin measures can give insight into the dynamics of social relationships: a review. *Front Behav Neurosci* 2014; **8**: 68.
12. Delorme A, Makeig S. EEGLAB: an open source toolbox for analysis of single-trial EEG dynamics including independent component analysis. *J Neurosci Methods* 2004; **134**: 9-21.
13. Pion-Tonachini L, Kreutz-Delgado K, Makeig S. The ICLabel dataset of electroencephalographic (EEG) independent component (IC) features. *Data Brief* 2019; **25**: 104101.
14. Cohen MX. *Analyzing neural time series data: theory and practice.* MIT Press: Cambridge, 2014.
15. Canolty RT, Edwards E, Dalal SS, Soltani M, Nagarajan SS, Kirsch HE et al. High gamma power is phase-locked to theta oscillations in human neocortex. *Science* 2006; **313**: 1626-1628.
16. Hülsemann MJ, Naumann E, Rasch B. Quantification of Phase-Amplitude

Coupling in Neuronal Oscillations: Comparison of Phase-Locking Value, Mean Vector Length, Modulation Index, and Generalized-Linear-Modeling-Cross-Frequency-Coupling. *Front Neurosci* 2019; **13**: 573.

17. Gertler J, Novotny S, Poppe A, Chung YS, Gross JJ, Pearlson G et al. Neural correlates of non-specific skin conductance responses during resting state fMRI. *NeuroImage* 2020; **214**: 116721.
18. Horstick J, Siebers S, Backhaus C. Examination of the skin conductance level (SCL) as an index of the activitx of the sympathetic nervous system for application in the continuous blood pressure measurement. Int J Biomed Eng Sci 2018; **5**: 1-11.
19. Schoormans D, Kop WJ, Kunst LE, Riem MME. Oxytocin effects on resting-state heart rate variability in women: The role of childhood rearing experiences. *Compr. Psychoneuroendocrinol* 2020; **3**: 100007.
20. Tracy LM, Gibson SJ, Labuschagne I, Georgiou-Karistianis N, Giummarra MJ. Intranasal oxytocin reduces heart rate variability during a mental arithmetic task: A randomised, double-blind, placebo-controlled cross-over study. *Prog Neuropsychopharmacol Biol Psychiatry* 2018; **81**: 408-415.
21. Rebollo I, Devauchelle AD, Béranger B, Tallon-Baudry C. Stomach-brain synchrony reveals a novel, delayed-connectivity resting-state network in humans. *Elife* 2018; **7**: e33321.
